# Renal Tissue Thawed for 30 Minutes Is Still Suitable for Gene Expression Analysis

**DOI:** 10.1371/journal.pone.0093175

**Published:** 2014-03-31

**Authors:** Yi Ma, Xiao-Nan Kang, Wen-Bin Ding, Hao-Zheng Yang, Ye Wang, Jin Zhang, Yi-Ran Huang, Hui-Li Dai

**Affiliations:** 1 Department of Biobank, Renji Hospital, School of Medicine, Shanghai JiaoTong University, Shanghai, China; 2 Department of Urology, Renji Hospital, School of Medicine, Shanghai JiaoTong University, Shanghai, China; 3 Department of Central Laboratory, Renji Hospital, School of Medicine, Shanghai JiaoTong University, Shanghai, China; TGen, United States of America

## Abstract

Some biosamples obtained from biobanks may go through thawing before processing. We aim to evaluate the effects of thawing at room temperature for different time periods on gene expression analysis. A time course study with four time points was conducted to investigate the expression profiling on 10 thawed normal mice renal tissue samples through Affymetrix GeneChip mouse gene 2.0 st array. Microarray results were validated by quantitative real time polymerase chain reactions (qPCR) on 6 candidate reference genes and 11 target genes. Additionally, we used geNorm plus and NormFinder to identify the most stably expressed reference genes over time. The results showed RNA degraded more after longer incubation at room temperature. However, microarray results showed only 240 genes (0.91%) altered significantly in response to thawing at room temperature. The signal of majority altered probe sets decreased with thawing time, and the crossing point (Cp) values of all candidate reference genes correlated positively with the thawing time (*p*<0.05). The combination of *B2M*, *ACTB* and *PPIA* was identified as the best choice for qPCR normalization. We found most target genes were stable by using this normalization method. However, serious gene quantification errors were resulted from improper reference genes. In conclusion, thirty minutes of thawing at room temperature has a limited impact on microarray and qPCR analysis, gene expression variations due to RNA degradation in early period after thawing can be largely reduced by proper normalization.

## Introduction

Today biological samples become an increasingly important tool for biomedical research into human diseases [1], [2]. High quality biosample with RNA closely representing the amount of transcripts in vivo can ensure a more accurate downstream molecular assay [3]–[5]. However, some pre-analytical factors like biosample collection, handling, or processing can affect the RNA quality [6]–[9]. Some fresh tissue handling protocols proposed that 30 minutes is the longest acceptable time period between surgical removal and freezing [10]. However in recent years, tissue samples often come from biobanks and may undergo thawing before being processed (e.g. RNA extraction). There is paucity of studies demonstrating the suitable time limit for thawing at room temperature within which DNA, RNA and protein analyses were not impacted.

Like fresh tissue samples, the quality of the frozen samples may also be affected by some pre-analytical factors like anesthesia, surgical manipulation, and transport mode. Additionally, some frozen tissue may go through thawing before being processed, and gene expression analysis in thawed tissue may be more subject to RNA degradation [11], [12]. A freeze-thaw cycle can rupture most cell structure and activate intrinsic RNase, resulting in RNA degradation which is believed to be one of the most important factors affecting gene expression [13]. Even housekeeping genes showed high variations in degraded RNA samples [14]. It was reported in quantitative real time polymerase chain reaction (qPCR) analysis that using reference gene with a short amplicon located closer to the 3′-end could remove variations for some targeted genes in degraded RNA samples [15], [16], but it is still unknown how tissue thawing affects gene expression profile, and there is still no common consensus on proper normalization genes in thawed tissue.

In this study we aim to evaluate the effects of thawing on gene expression analysis, as measured by microarray analysis and qPCR method. In addition, we searched for the suitable housekeeping genes which were most stable in thawing process for qPCR normalization.

## Materials and Methods

### Ethics Statement

We performed all animal surgery under ketamine anesthesia, and took all efforts to minimize animal suffering. All procedures were reviewed and approved by the ethics committee of Renji hospital.

### Tissue Collection and Processing

In order to exclude some pre-analytical factors like anesthesia, operation or transport time in human tissue samples, normal renal tissue was obtained from ten C57BL/6 10-week-old mice and each was immediately sectioned into four aliquots. All the samples were snap-frozen and then kept at −80°C for two weeks. The first aliquot of each sample was immediately placed in Trizol reagent (Invitrogen, Carlsbad, CA, USA), while the remaining three pieces were kept at room temperature for 5, 15, 30 min, respectively, before RNA extraction.

### RNA Extraction, RNA Quality Evaluation and cDNA synthesis

The total RNA was isolated with Trizol reagent from about 50 mg renal tissue according to manufacturer's instructions. The RNA yields and A260/A280 ratios were measured by NanoDrop ND-2000c Spectrophotometer (NanoDrop Technologies, Montchanin, DE, USA). RNA integrity number (RIN) was detected using pooled RNA samples. The isolated RNA samples at each time point were pooled and analyzed on the Agilent 2100 bioanalyzer (Palo Alto, CA, USA) using RNA 6000 Nano LabChip kit (Agilent Technologies, Santa Clara, CA, USA). RNA integrity was evaluated by Agilent software and had values ranging from 1–10, with 1 being the most degraded and 10 being the most intact. RIN value was obtained from an artificial neuron network prediction model based on feature selection from the electropherograms: the total RNA ratio, 28S peak height, 28S area ratio, fast area ratio, and so on [17]. Each isolated RNA sample (one microgram) was reverse transcribed using the Primescript RT reagent kit with gDNA Eraser (Takara bio Inc, Japan) according to manufacturer's instructions. Briefly, a DNase digestion was performed at 42°C for 2 min, followed by the reverse transcriptase reaction conducted at 37°C for 15 minutes and the enzymes were inactivated at 85°C for 5 seconds. The cDNA samples were stored at −20°C.

### Microarray analysis and qPCR validation

RNA samples of each time point were pooled and converted to labeled single-stranded DNA according to the GeneChip whole transcript terminal labeling user manual (Ambion wt expression kit) and hybridized to an Affymetrix GeneChip mouse gene 2.0 st array on GeneChip hybridization oven 640 (Affymetrix, USA). The staining, washing and scanning of the arrays were performed on a GeneChip fluidics 450 workstation (Affymetrix, USA) and GeneChip scanner 3000 7G (Affymetrix, USA) according to manufacturer's instruction. Gene expression data was analyzed using Partek genomic suite (Partek, USA). Robust Multichip Analysis (RMA) algorithm was used for normalization to remove artifactual differences between arrays and make the distribution of probe intensities the same for every chip. We used the Gene Ontology (GO) database to interpret the differentially expressed gene data set. The array data has been submitted to ArrayExpress (Accession number: E-MTAB-2169). We also did a verification analysis on the genes in relation to renal cancer based on searching NCBI (http://www.ncbi.nlm.nih.gov/gene/) with the keywords “renal cancer”, “renal cell cancer”, “renal cell carcinoma”, “kidney cancer”. This search identified 343 genes in *Mus musculus*.

All the 40 cDNA samples were amplified by LightCycler 480 Real-Time PCR system (Roche Diagnostics, Mannheim, Germany) using the SYBR premix ex taq (Takara bio inc, Japan) according to manufacturer's instructions for 4 groups of genes: most frequently used reference genes in renal tissue: *ACTB, B2M, GAPDH, HPRT, PPIA, TBP*; significantly altered genes found in microarray results: *ANGPT2, KLRA9, FABP7*; hypoxia related genes: *HIF1A, HMOX1, VEGFA*; and renal cell carcinoma (RCC) related genes: *CAR9, CD274, MKI67, PTEN, VHL*. (Table 1) All the four aliquots from one tissue sample were always analyzed in the same PCR run, to minimize the between-run variations. Water instead of cDNA was used as the negative control. We assessed PCR repeatability (intra-assay) and reproducibility (inter-assay) by pooled cDNA obtained from the whole set of samples. The qPCR mixture was prepared in a final volume of 30 µL, including 15 µL Taq DNA polymerase, 0.6 µL forward primer (10 µmol/L), 0.6 µL reverse primer (10 µmol/L), 1.5 µL cDNA samples, and 12.3 µL dH_2_O. The two-step qPCR was: denaturation at 95°C for 30 seconds, followed by 45 cycles of 95°C for 5 seconds and 60°C for 20 seconds. For each candidate reference gene, serial dilution of cDNA sample was used to make a standard curve for qPCR and amplification efficiency was calculated according to the slope of the curve.

**Table 1:** Sequences and related information of primers.

Gene symbol	Gene name	Accession No.	Primer Sequence [5′→3′] *	Amplicon length
*ACTB*	Actin, beta	NM_007393	F: CATCCGTAAAGACCTCTATGCCAAC	171
			R: ATGGAGCCACCGATCCACA	
*ANGPT2*	Angiopoietin 2	NM_007426	F: CCTCGACTACGACGACTCAGT	146
			R: TCTGCACCACATTCTGTTGGA	
*B2M*	beta-2-microglobulin	NM_009735	F: TGCTACTCGGCGCTTCAGTC	200
			R: AGGCGGGTGGAACTGTGTTAC	
*CAR9*	Carbonic anhydrase 9	NM_139305	F: CAACCCTTGAATGGGCGAAC	145
			R: CGATGCTGGTGACAGCAAAGA	
*CD274*	CD274 antigen	NM_021893	F: AGCGAATCACGCTGAAAGTCAA	109
			R: GGATAACCCTCGGCCTGACATA	
*FABP7*	Fatty acid binding protein 7	NM_021272	F: GATCAATTTCCAGCTGGGAGAAGAG	178
			R: CATAACAGCGAACAGCA	
*GAPDH*	Glyceraldehyde-3-phosphate dehydrogenase	NM_008084	F: TGTGTCCGTCGTGGATCTGA	150
			R: TTGCTGTTGAAGTCGCAGGAG	
*HIF1A*	Hypoxia inducible factor 1	NM_010431	F: GGACGATGAACATCAAGTCAGCA	146
			R: AGGAATGGGTTCACAAATCAGCA	
*HMOX1*	Heme oxygenase (decycling) 1	NM_010442	F: TGCAGGTGATGCTGACAGAGG	144
			R: GGGATGAGCTAGTGCTGATCTGG	
*HPRT1*	Hypoxanthine guanine phosphoribosyl transferase 1	NM_013556	F: TTGTTGTTGGATATGCCCTTGACTA	189
			R: AGGCAGATGGCCACAGGACTA	
*KLRA9*	killer cell lectin-like receptor subfamily A, member 9	NM_010651	F: AGATTCCTCACGGGACACAG	187
			R: TGGAATAACATGGCGTTGAA	
*MKI67*	Antigen identified by monoclonal antibody Ki 67	NM_001081117	F: GATGAGCCTGTGAGGCTGAGAC	150
			R: TCTTGAGGCTCGCCTTGATG	
*PPIA*	Peptidylprolyl isomerase A	NM_008907	F: ATCTTGTCCATGGCAAATGCTG	146
			R: AAACGCTCCATGGCTTCCAC	
*PTEN*	Phosphatase and tensin homolog	NM_008960	F: GGACGGACTGGTGTAATGATTTG	196
			R: GCAGTGCCACGGGTCTGTAA	
*TBP*	TATA box binding protein	NM_013684	F: CATTCTCAAACTCTGACCACTGCAC	161
			R: CAGCCAAGATTCACGGTAGATACAA	
*VEGFA*	Vascular endothelial growth factor A	NM_001025257	F: ACATTGGCTCACTTCCAGAAACAC	108
			R: TGGTTGGAACCGGCATCTTTA	
*VHL*	Von Hippel-Lindau tumor suppressor	NM_009507	F: AACGGAGCTGTTTGTGCCATC	111
			R: AGGCTCCGCACAACCTGAA	

F: Forward R: Reverse.

### Data analysis

Statistical analyses were performed with the Statistical Package for Social Science (SPSS), version 16.0 (SPSS, Chicago, IL, USA). All tests were two-tailed, and *p*<0.05 was considered significant. In microarray data analysis, we set fold change >2 or <0.5 as cutoff values to detect differentially expressed genes. Data of probe sets was log transformed with base 2 (log_2_), log_2_ ratios were calculated in each thawed sample relative to the 0-time signal. The log_2_ ratio data for the filtered sets was displayed by clustering (Gene Cluster 3.0) and plotting the heatmap (Java TreeView, 1.1.4r3). We plotted qPCR data by SigmaPlot, version 11.0 (Systat Software, San Jose, CA, USA). Normfinder (version 20) [18] and geNorm plus programs (version 2.1) [19] were used to search for the most stable housekeeping genes according to developers' manual. GeNorm plus takes into account the PCR efficiencies of the candidate reference genes. For both programs, the lower the value, the better the gene stability.

## Results

**Figure 1:**
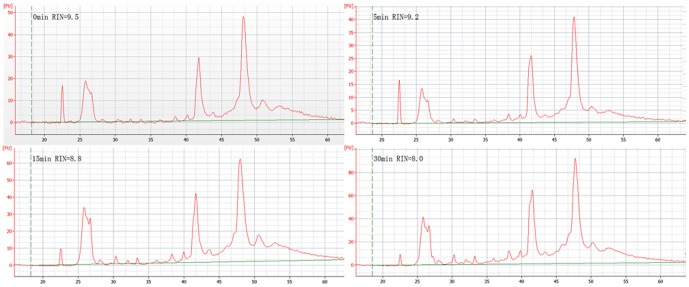
RIN values of the renal tissue samples thawed for different time periods at room temperature.

All the A260/A280 ratios of isolated RNA samples were >1.95. RIN values of the four pooled samples decreased with thawing time (0 min: 9.5; 5 min: 9.2; 15 min: 8.8; 30 min: 8.0) (Fig. 1), which indicated a measureable RNA degradation in thawed tissue, although all the RIN values were in the range of >6 which was recommended as a threshold for high and low quality RNA samples [17].

**Figure 2:**
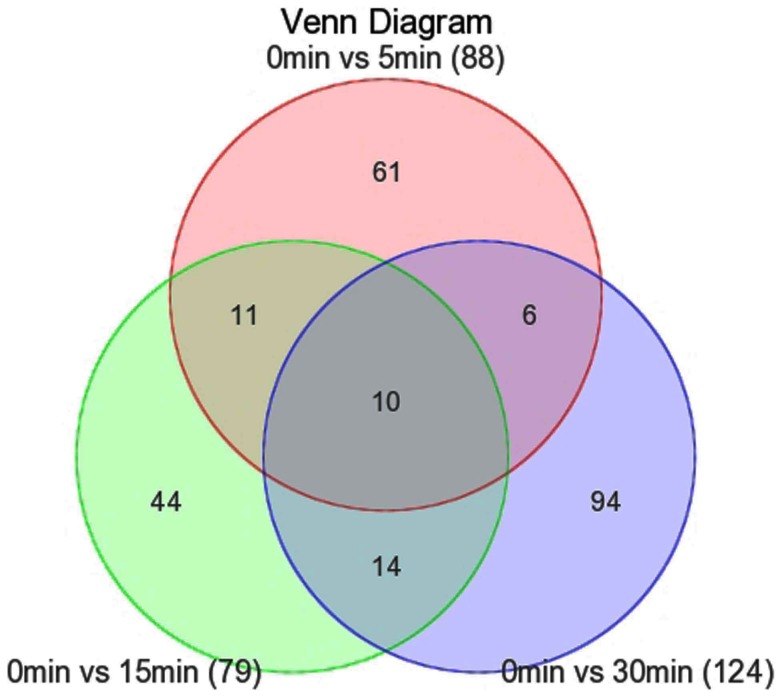
Venn diagram demonstrating significantly altered gene numbers between 0–5 min; 0–15 min; and 0–30 min.

**Figure 3:**
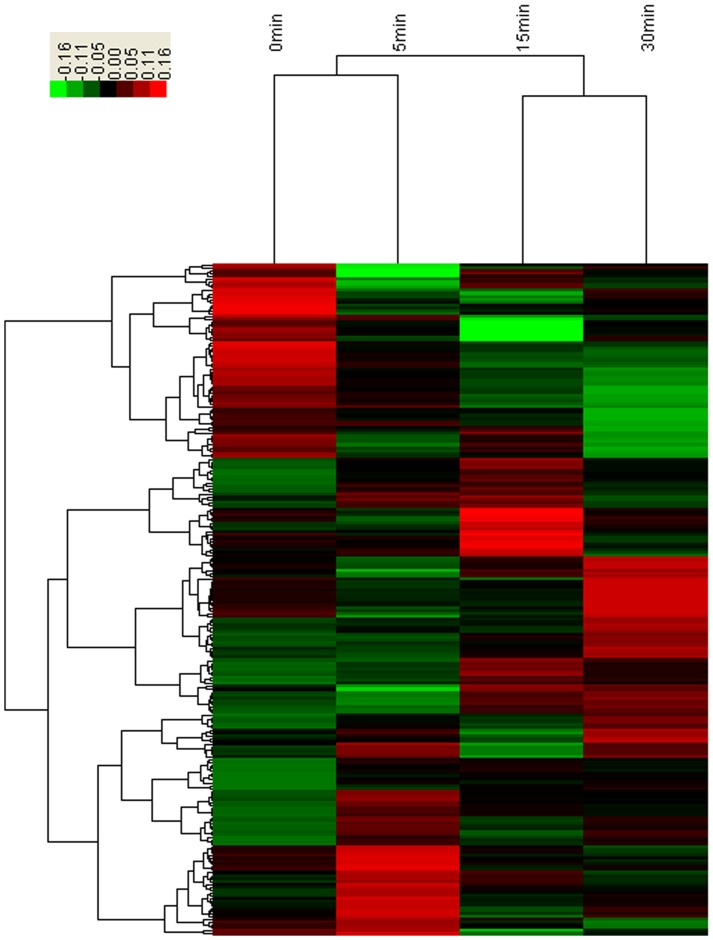
Hierarchical clustering of differentially expressed genes at the four time points after tissue thawing. Log_2_ ratios were calculated for each sample at the time points 5 min, 15 min, and 30 min relative to the 0-time, and then the ratios were mapped on a green (−0.16) to red (0.16) color scale.

The mouse gene 2.0 st array interrogates a total of 35,240 reference sequence transcripts with 26,515 gene count. Across the time course, a total of 240 genes (0.91%) changed significantly due to thawing in at least one of the aliquots at 5 min, 15 min, and 30 min compared to 0 min. At the later 3 time points, there were 88 genes, 79 genes, and 124 genes considerably up- or down-regulated respectively (Fig. 2) and we listed all these genes in Table S1. Figure 3 also showed the log_2_ ratio data of the filtered sets by heat map and hierarchical clustering. The signal of most probe sets decreased with thawing time in response to RNA degradation. The 240 significantly altered genes covered a wide range of biological processes by searching GO database, the most represented categories were “cell surface receptor linked signal transduction” (87 genes), “G-protein coupled receptor protein signaling pathway” (85 genes), and “neurological system process” (68 genes). We also carried out a verification analysis on the genes in relation to renal cell cancer. Verification on the gene lists can help to assess if RNA degradation in response to thawing affects the performances of these crucial genes. The results demonstrated none was identified in relation to renal cell cancer.

**Figure 4:**
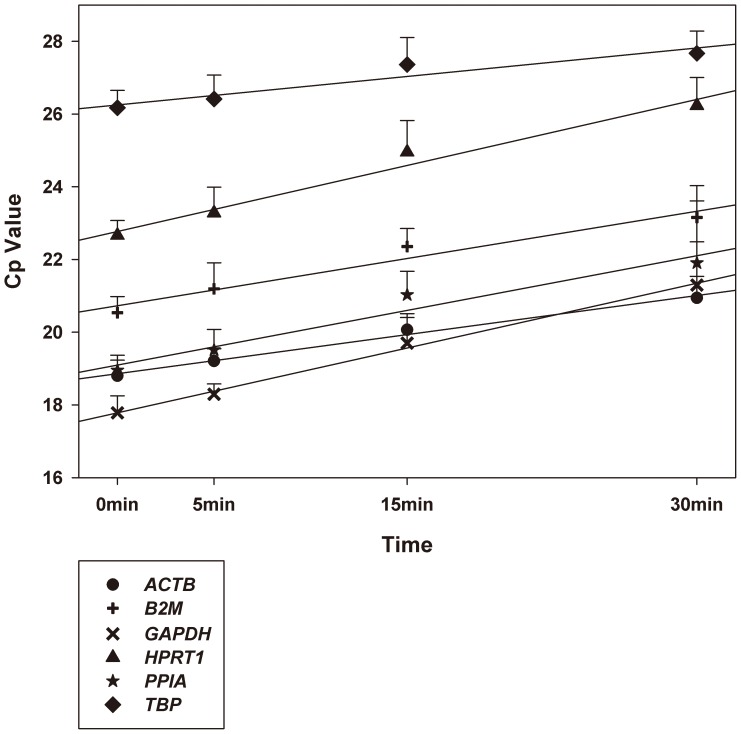
Linear regression analysis between Cp values and thawing time and comparison among 6 candidate reference genes in thawed renal tissue samples. Whiskers illustrate the standard deviation of Cp values. N = 10 at each time point. The regression coefficients of *ACTB*, *B2M*, *GAPDH*, *HPRT1*, *PPIA*, and *TBP* are 0.072, 0.087, 0.119, 0.121, 0.100, and 0.052, respectively. All the *p* values <0.05.

For the six candidate reference genes in qPCR analysis, the intra-run coefficients of variation ranged from 0.23 to 0.61%, and the inter-run variations ranged from 0.82 to 2.31%. Crossing point (Cp) (also defined as Ct value) values of each reference gene were similar among different samples with the same delayed time which indicated the inter-sample gene expression variability was low (standard deviation of all the Cp values of six reference genes were smaller than one, data not shown). Cp values of all candidate reference genes correlated positively with the time of thawing at room temperature. (All the *p* values<0.05) Figure 4 showed the linear regressions between Cp values and thawing time. All the regression coefficients were >0, and all the *p* values <0.05.

**Figure 5:**
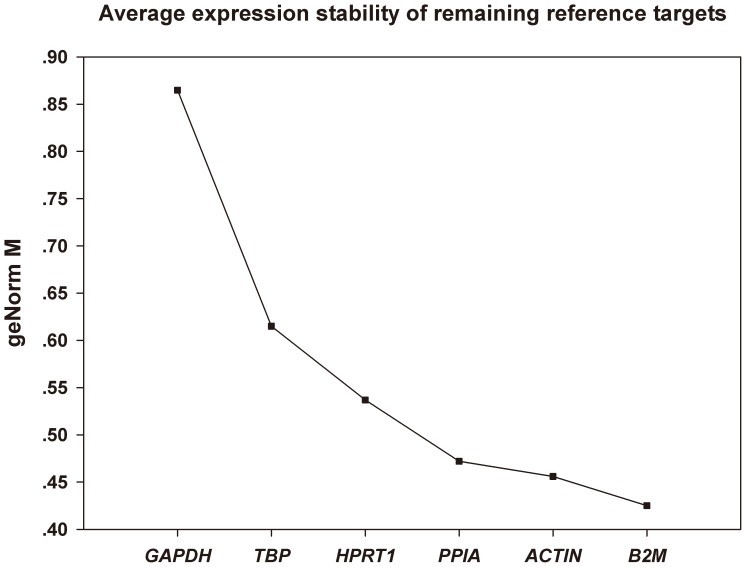
Average expression stability values of 6 candidate reference genes according to geNorm plus algorithm at the 4 time points.

**Figure 6:**
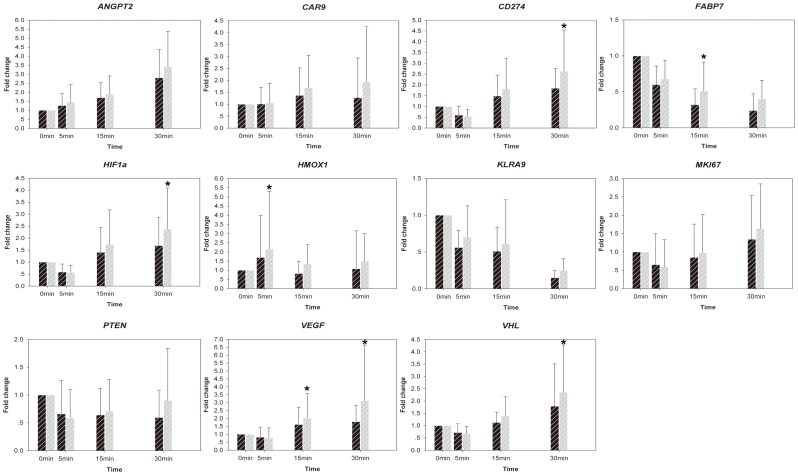
Mean relative expression levels of 11 target genes based on normalizations with *GAPDH* (brown) or the combination of *B2M*, *ACTB* and *PPIA* (black). Normalization with *GAPDH* lead to increased relative expression levels for most of the target genes at the time points 5 min, 15 min, and 30 min comparing with the 0-time point. (N = 10 at each time point) Whiskers illustrate the standard deviation of Cp values. Fold change >2 or <0.5 was considered significant. *: Significant differences were found between the two normalization methods.

Expression stability of the 6 candidate reference genes at the 4 time points was tested by Normfinder and GeNorm plus. Both programs consistently reported that *B2M* was the most stable reference gene in 30 min, while *GAPDH* was ranked last. GeNorm plus also provided a rank order of the six reference genes (Fig. 5). *B2M*, *ACTB* and *PPIA* were the best combination for normalization reported by geNorm plus. We then compared the relative expression level of 11 target genes in the degraded renal samples based on different normalization (Fig. 6). The 11 target genes included 1 up-regulated gene, 2 down-regulated genes and 8 unchanged genes demonstrated by microarray data. Figure 6 showed that when gene expression levels were normalized with the combination of *B2M*, *ACTB* and *PPIA*, the results were consistent with microarray's. However, normalization with *GAPDH* led to increased relative expression levels of most target genes compared with the 0 min and five unchanged genes in microarray data changed significantly in qPCR analysis based on *GAPDH* normalization. (*CD274, HIF1A, HMOX1, VEGFA, VHL*).

## Discussion

There have been some time course studies evaluating gene variation in surgically removed tissue before biobanking [20]–[22], and some other studies compared fresh frozen tissue with fresh tissue by microarray or RNA quality evaluation [23], [24]. In this study, we instead evaluated the impact of different time periods between thawing and processing on gene expression profiling. It was reported that thawed tissue should be immediately processed with denaturing solutions to acquire a credible gene expression profiling data, however we found only limited changes in relative gene expression levels within 30 min after thawing. The results of microarray analysis showed that only 240 from 26,515 genes were up- or down-regulated substantially within this period, in which none was associated with renal cancer. The gene variations were also validated by carefully normalized qPCR and the results were consistent with microarray data. Because only a few genes were modulated considerably in relative expression level, we believed renal tissue thawed for 30 min is still suitable for gene expression analysis, with proper normalization.

It was demonstrated that partially degraded RNA samples are tolerated by microarrays with 3′-biased probe selection designs [12], [25]. Mouse gene 2.0 st array used in this study is more accurate and more sensitive than 3′-biased expression array designs, since each of the detected transcript is represented by a median of 22 unique probes spread across the full length of the transcript. Even though, only 240 differently expressed genes were identified in this study. Our results suggest that slightly to moderately degraded renal tissue can still be used in microarray and qPCR studies, and gene expression variations in early period after thawing can be largely reduced by proper normalization. The appropriate selection of a normalization gene is critical in relative quantification especially in degraded biosamples [26], because even housekeeping genes show high variations, and some of the reference genes are even more fragile to degradation [27]. Several studies have suggested using carefully selected normalization genes in qPCR for the purpose to remove the nonspecific variations in degraded RNA samples [14], [28], [29]. In this study, we evaluated six most frequently used reference genes in thawed renal tissue by using Normfinder and geNorm plus algorithms, and we found *B2M* or the combination of *B2M*, *ACTB* and *PPIA* were the best normalization method in thawed kidney tissue. Normalization with inappropriate reference genes may lead to significantly different results in gene expression levels which were also inconsistent with microarray results.

Although slight RNA degradation was found within 30 min and all the RIN values were still in the high quality range, Cp values of nearly all the six reference genes showed the same trends towards increasing along with thawing time by linear regression analysis. The results indicated housekeeping genes degraded in thawed tissue within 30 min. This is in accordance with the study conducted in thawed tonsil tissue by Botling and colleagues, who also found even minimally decreased RNA quality in thawed tissue may result in significant changes in gene expression [15]. In this study, the Cp values of some genes increased less than that of reference genes, resulting in a higher relative expression level. In contrast, the Cp values of some genes increased more rapidly than that of reference genes resulting in decreased expression values. Most genes in this study showed an increased Cp values that paralleled the reference genes and consequently the expression levels did not change considerably along with thawing time. As a result, we can find when examining relative expression levels in thawed tissue, it is critical to select most representative reference genes for normalization, an inappropriate reference may cause significantly misleading results.

Modulated genes in thawing process could be up- or down-regulated, but most genes were down-regulated owing to RNA degradation which indicated the RNA reduction was the main event in thawing tissue. As for the up-regulated genes, one possible explanation might be that the normalization genes degraded more rapidly than those up-regulted genes in thawed tissue, resulting in higher relative expression level. Secondly, warm ischemia stress in thawing process induced cell metabolic activity [30], such as survival or apoptotic process, resulting in changes in the transcript levels. In this study we found the relative expression amounts of stimulus response genes (*OLFR* gene family) and cellular defense response genes (*DEFB* gene family) demonstrated significant increase within 30 min. Some studies have also reported in fresh tissue that the stress response genes consistently increased at the early stage after surgical removal, in response to warm ischemia [8], [22], [31], [32]. In this study, although a freeze-thaw cycle was performed, a portion of cells in the tissue may still survive and metabolic activity induced by warm ischemia may lead to up-regulated expression of some genes.

The present study may be subject to several limitations. First, the study is limited to the small sample size. Second, we analyzed microarray and qPCR data only within the time period 30 min, we didn't know the assay results if samples were thawed for more than 30 min. However, we believe this time period is long enough for the thawed tissue to be processed. Third, we measured only 6 reference genes and it is possible some excluded genes may be more stable than *B2M* in thawed renal tissue. However all the 6 genes were most frequently used in renal tissue, we excluded the rarely used genes due to the limited tissue material available. Besides, qPCR normalization method can't prevent all the false positive or negative results, fresh frozen tissue without thawing is always better than thawed tissue.

## Conclusion

Thirty minutes of tissue thawing at room temperature led to a slight loss of RNA integrity and has a limited impact on microarray and qPCR analysis. Gene expression variation caused by RNA degradation in early period after thawing can be largely reduced by proper normalization. Housekeeping genes should be carefully selected in degraded samples for validation of microarray data; *B2M* or the combination of *B2M*, *ACTB* and *PPIA* are the most suitable selections for normalization in thawed renal tissue samples. It was difficult to prevent the misleading research results when selecting invalidated reference genes for normalization.

## Supporting Information

Table S1 **Differentially expressed genes at the time points 5 min, 15 min and 30 min compared with 0 min.**
(XLSX)
